# Turning dread into capital: South Africa’s AIDS diplomacy

**DOI:** 10.1186/1744-8603-9-8

**Published:** 2013-03-05

**Authors:** Pieter Fourie

**Affiliations:** 1 Department of Political Science, Stellenbosch University, Private Bag X1, 7602, Matieland, South Africa

**Keywords:** HIV, AIDS, South Africa, Niche diplomacy, Foreign policy, Middle power

## Abstract

**Background:**

In much of the world, President George W. Bush was not admired for his foreign policy and diplomacy. It is therefore ironic that Bush’s single most uncontested foreign policy triumph was an instance of what has now become known as “health diplomacy”. In 2003 Bush launched the President’s Emergency Plan for AIDS Relief, a five-year $15 billion initiative to fight HIV/AIDS, mostly in Africa. The president’s pragmatic health diplomacy may well save his foreign policy legacy. This article argues that a middle power such as South Africa should consider a similar instrumental AIDS diplomatic strategy, to rehabilitate its public health as well as foreign policy images.

**Discussion:**

This article reflects on the emergence and contemporary practice of health diplomacy. In particular, it explores the potential of niche areas within health diplomacy to become constructive focal points of emerging middle powers’ foreign policies. Middle powers often apply niche diplomacy to maximise their foreign policy impact, particularly by pursuing a multilateral agenda. The literature on middle powers indicates that such foreign policy ambitions and concomitant diplomacy mostly act to affirm the global status quo. Instead, this paper argues that there may well be niches within health diplomacy in particular that can be used to actually challenge the existing global order. Emerging middle powers in particular can use niche areas within health diplomacy in a critical theoretical manner, so that foreign policy and diplomacy become a project of emancipation and transformation, rather than an affirmation of the world as it is.

**Summary:**

The article first describes the emergence and contemporary practice of health diplomacy; this is followed by a discussion of niche diplomacy, in particular as it applies to the foreign policy agendas of emerging middle powers. It then reviews South African foreign policy and diplomacy, before situating these policies within the context of emerging mechanisms of south-south multilateralism. The article concludes by synthesizing these elements and advocating for a South African AIDS diplomacy, emphasizing its potential to galvanize a global project of emancipation.

## Background

When the Group of Eight (G-8) industrialized countries decided at their 2001 meeting in Genoa to establish the Global Fund to Fight AIDS, Tuberculosis, and Malaria to increase international funding for interventions against the three diseases, the United States was a leading supporter. The fund was a public-private partnership set up in 2002 with formal status as a foundation under Swiss law. In the fund’s first two years, the United States accounted for nearly half the total amount pledged and challenged other donors to increase their contributions. By 2008 the Global Fund had committed $15.6 billion to AIDS activities in 140 countries [1].

Additionally, in 2003 U.S. president George W. Bush launched the President’s Emergency Plan for AIDS Relief (PEPFAR), a five-year, $15 billion initiative to fight HIV/AIDS, mostly in Africa. PEPFAR has been called the largest initiative ever undertaken by a single country to address a disease, and because of the amount of resources it provided, the U.S. government strengthened its position as a major funder of AIDS activities in the global south. By the end of 2008, the program had provided treatment to two million people in 15 focus countries, 12 of which were African [2].

Despite these actions, it should be recalled that in much of the world Bush was not admired for his foreign policy and diplomacy. Because he chose not to follow the liberal institutional norms and mechanisms that had been established since World War II, Bush was accused of practicing a diplomacy of circumvention. It is therefore ironic that Bush’s single most uncontested foreign policy triumph was an instance of what has now become known as “health diplomacy.” Both PEPFAR and the Global Fund are exemplars of a radical shift in global health governance that may well save Bush’s foreign policy legacy.

The practice of health diplomacy is not limited to major powers such as the United States, however. Niche areas of health diplomacy have the potential to become constructive focal points of emerging middle powers’ foreign policies as well. Indeed, middle powers often apply niche diplomacy to maximize their foreign policy impact, particularly by pursuing a multilateral agenda. Although such foreign policy ambitions and concomitant diplomacy mostly act to affirm the global status quo, there may well be niches within health diplomacy in particular that can be used to challenge the existing global order. Emerging middle powers can use these niche areas within health diplomacy as tools for challenging and working to change the status quo so that foreign policy and diplomacy become a project of emancipation and transformation, rather than an affirmation of the world as it is. At the same time, niche areas within health diplomacy can serve a different, domestic role: to make explicit and to calibrate emerging middle powers’ foreign policy priorities.

South Africa is an example of a young democracy that seems to have lost its way in how it considers and practices diplomacy it is what one observer has called a “rogue democracy” [3]. On paper, the country’s foreign policy is aspirational, idealistic, and fairly typical of an emerging middle power, but its actions have been criticized as inconsistent, confusing, and even sinister. In the same way that South Africa transformed its horrific history of racism and apartheid to become a beacon of human rights internationally, however, it can transform its appalling AIDS burden to take a global lead on this issue. AIDS diplomacy can serve as a thematic hub or *leitmotif* around which the aspirational elements of South African foreign policy can be aligned and operationalized. Moreover, South African AIDS diplomacy can be used to drive a transformationalist global agenda not only on issues of global health governance, but in terms of global and multilateral reform more broadly if pursued thoughtfully.

This paper first describes the emergence and contemporary practice of health diplomacy, followed by a discussion of niche diplomacy, in particular as it applies to the foreign policy agendas of emerging middle powers. It then reviews South African foreign policy and diplomacy before situating these policies within the context of emerging mechanisms of south-south multilateralism. The paper concludes by synthesizing these elements and advocating for South African AIDS diplomacy, emphasizing its potential to galvanize a global project of emancipation.

## Discussion

### The emergence of health in foreign policy

In September 2006 the foreign ministers of Brazil, France, Indonesia, Norway, Senegal, South Africa, and Thailand launched the Global Health and Foreign Policy Initiative, and on March 20, 2007, they issued the Oslo Ministerial Declaration, which defines global health as a pressing foreign policy issue:

In today’s era of globalisation and interdependence there is an urgent need to broaden the scope of foreign policy. Together, we face a number of pressing challenges that require concerted responses and collaborative efforts. We must encourage new ideas, seek and develop new partnerships and mechanisms, and create new paradigms of cooperation. *We believe that health is the most important, yet still broadly neglected, long-term foreign policy issue of our time*. […] We have therefore agreed to make impact on health a point of departure and a defining lens that each of our countries will use to examine key elements of foreign policy and development strategies, and to engage in a dialogue on how to deal with policy options from this perspective [4]. [Emphasis added]

The declaration states that its aim is to harness the benefits of globalization, and it identifies an agenda that includes, but is not limited to, the following actions:

• governing global health security, focusing on preparedness in foreign policy, the establishment of global surveillance mechanisms to identify epidemiological threats to collective security, and the timely and concerted mobilization of resources to combat such threats;

• identifying and addressing the current shortage and maldistribution of trained health workers;

• aligning action in cases of natural disasters and other crises;

• responding in a concerted fashion to the global AIDS pandemic in particular;

• combating climate change and the negative impacts it has on global health distribution;

• applying a health perspective to identify development priorities, the allocation of aid and resources, and the structural barriers to greater material equity and justice in the world;

• establishing trade policies and measures to implement and monitor agreements, particularly with regard to pharmaceutical access; and

• developing the global health governance architecture, bilaterally, regionally and multilaterally.

Although the agenda for action and the aspirations for improved global health outcomes are not new, the emphasis on linking health so explicitly to foreign policy certainly is. The declaration shows that health is an issue that can initiate dialogue and cooperation across borders, thus building trust among state and non-state actors.

Shortly after the Oslo Declaration was issued, the head of the World Health Organization (WHO) and the foreign ministers of France and Norway issued a word of caution: for health to be a sustainable lens for foreign policy thinking and agenda setting, it must be mainstreamed into the training of diplomats. In addition, they urged that an overemphasis on health *security* concerns not overshadow the opportunity afforded by the Oslo Declaration to use health as a constructive and novel perspective to shape international, transnational, and global action [5].

The latter is an important caveat, as the history of global health is often more closely associated with the governance of health threats than with the search for equity, justice, and well being [6]. For instance, the first International Sanitary Conference, in 1851, focused on combating the spread of cholera, plague, and yellow fever. Health diplomacy as it was then practiced focused mainly on negotiating international disease control conventions, the first one of which was concluded in 1903. The International Organization for Public Hygiene was established in 1907, but its role was to protect international commerce from the possibility of free-riding epidemics. Gradually the architecture of international health governance expanded, culminating in the establishment of the WHO in 1948—almost as an afterthought to the creation of the UN family. Yet, the International Health Regulations (IHR) drawn up through this body remain to this day mainly concerned with surveillance and the threat of global epidemics to collective security. This mindset reflects the broad Realist definition of diplomacy: “the official means by which one state formally relates to other states … [as] a mechanism—one among many—used in furtherance of the national interest” [7].

It is not surprising, then, that early in the last decade, and especially after the 9/11 attacks on the United States, policymakers came to view infectious diseases as threats to global security and stability. AIDS in particular was seen as a proxy for the cause of potential state fragility in the developing world, which in turn was conceptualized as the main driver of global terrorism. Over the last few years, however, it has become clear that AIDS and other diseases are not the *cause* of global insecurity and state failure; rather, domestic and multilateral policy interventions should focus on the real drivers of the epidemic, social determinants of health. Despite this shift in thinking about the global AIDS pandemic, policymakers nevertheless tend to focus on the “high politics” of health issues that is, their security aspects rather than on “low politics” in which health issues are seen as a reflection of human dignity [8].

The Oslo Declaration warns that equating health diplomacy with health security would underestimate the emergence and limit the agenda of a multitude of new actors and resources devoted to the governance of global health. By 2007 the Bill and Melinda Gates Foundation’s budget surpassed that of the WHO, and the Foundation is now a member of a new elite of global health governance the so-called Health-8 (H-8) that also includes the WHO, the World Bank, the GAVI Alliance, United Nations Children’s Fund (UNICEF), United Nations Population Fund (UNFPA), UNAIDS, and the Global Fund. The emphasis of global health governance and diplomacy is on cultivating partnerships among state and non-state actors acting transnationally. This reflects the definition of health diplomacy as offering “new mechanisms to implement ambitious global health initiatives while at the same time securing favorable perceptions in a changing diplomatic space” [9].

The rapid increase in the number of actors and issues on the global health agenda has galvanized the search for greater alignment and concerted actions, with the Paris Declaration on Aid Effectiveness in 2005 being one example of a broader effort to reduce waste and duplication [10]. This push has become even stronger in light of the reduction in funding available for global public health, due to the impact of the ongoing global financial crisis. A consensus is developing that the focus of low- and middle-income countries in particular should be on strengthening health systems. Global health funding, for now, seems to have flatlined [11], exacerbating the need for improved global health governance, innovative health diplomacy, and better coordination among those who matter, about what matters and how to distribute the decreasing resources available. Since 2003, the South Africa’s support from PEPFAR has been billions of Rands, and during a visit to the United States in mid-2012 Deputy President Kgalema Motlanthe appealed to the American government not to reduce their financial support for AIDS treatment and prevention programmes in South Africa. As a result of this visit, the South African government has received assurances that PEPFAR funding will continue for another two years, although it is then very likely to decline significantly [12].

### Niche diplomacy

Those states and other actors that have been most prominent and successful at health diplomacy seem to be the ones that are best able to prioritize and focus their efforts. Former Australian foreign affairs minister Gareth Evans notes that less powerful states should undertake “niche diplomacy, which involves concentrating resources in specific areas best able to generate returns worth having, rather than trying to cover the field” [13]. In the practice of diplomacy an actor should not try to be all and do all for everyone. Unfocused and ineffectual diplomacy is frequently the consequence of lofty foreign policy documents that are conceptualized in such broad and abstract terms that they have no sustainable traction in their application.

Even very powerful states understand the importance of selecting specific priorities when they practice health diplomacy. For instance, during the second half of the Bush administration, the secretary of Health and Human Services, Michael Leavitt, made it clear that the attainment of soft power—that is, cooption by persuasion rather than coercion—would be the aim of American medical diplomacy and humanitarian efforts. For Leavitt, public diplomacy—appealing directly to the population of other states—was a key element of public relations and a goal in itself: “I have heard HIV/AIDS victims in distant villages of Africa say the words ‘U-S-A’ with their lips and ‘thank you’ with their eyes” [14]. Of course, self-interest is never far away; Leavitt goes on to say that “[s]oft power builds trust for moments when hard power is required” [15]. Using health diplomacy to attain soft power has hardly been a strategy exclusive to the Bush presidency or the Republican party, however. The idea of “medical diplomacy” was introduced in 1978 by the special assistant to the president for health issues during the Carter administration [16].

Developing countries have also benefited from identifying niches in a more focused health diplomacy; Brazil is a key example. In 1988 Brazil formally recognized the right to health in its constitution, and soon thereafter it made health a central theme of its foreign policy actions. When antiretroviral drugs (ARVs) became available for the treatment of HIV/AIDS in 1996, the Brazilian government used its health-rights emphasis to increase access to these pharmaceuticals in order to provide universal treatment to all HIV-positive people in the country. Brazil cultivated transnational issue coalitions with NGOs as well as other states in the global south and granted compulsory licences for the local production of generic ARVs. Large pharmaceutical companies in the global north were outraged; some of the actions that Brazil pursued were illegal (or deemed as such) within the context of the Agreement on Trade-Related Intellectual Property Rights (TRIPS) in the newly established World Trade Organization (WTO). Nevertheless, Brazil’s transnational coalition won the day. In the process, Brazil became the first developing country to offer free ARV treatment to people living with HIV, despite claims by the World Bank that such a policy was not cost-effective [17]. More recently, Brazil has also played a key role in negotiating the Framework Convention on Tobacco Control (FCTC), using soft power to achieve its goals by serving as an example for domestic tobacco control, engaging in coalition politics, and providing leadership during the negotiation process [18].

Other developing states have selected a more focused medical diplomacy to achieve their foreign policy goals. Cuba has prioritized south-south links and seeks to build non-Western coalitions to foster a broader “anti-imperialist” global agenda. Although such language seems quite dated in the contemporary world, Cuba has been remarkably successful at achieving its southern solidarity by training and/or providing health personnel as its specific niche within health diplomacy. It should be noted, of course, that Venezuela foots the bill for most of Cuba’s medical largesse [19].

Finding a niche within the broad field of diplomacy—and even within health diplomacy more closely—seems to have more easily attainable outcomes for less powerful states. Such states may have “technical and entrepreneurial leadership on the world stage on specific issues” [20]. This is also a particular feature of middle powers, which may not seek power for their own sake, but instead act as “norm entrepreneurs” on issues where they have existing credibility. South Africa is a case in point: the country managed to survive its racist and divisive past and, after it democratized, in 1994 President Nelson Mandela went to work to establish the country as a symbol of nonracialism and reconciliation. South Africa achieved a level of credibility and stature by negotiating a peaceful transition, setting up a Truth and Reconciliation Commission, and establishing one of the most progressive liberal constitutions in the world. Mandela and his successors have capitalized on this credibility to project South African influence on the multilateral stage.

Middle powers such as Brazil, India, and South Africa also use transnational issue networks and links with global social movements to achieve their foreign policy goals. The case of Brazil in the WTO is an example of this; so too is South Africa and the Treatment Action Campaign (TAC). In the late 1990s South Africa lobbied with Brazilian activists and members of civil society in the global north to successfully challenge large pharmaceutical companies’ insistence that the country should not be allowed to parallel import generic medications. As one analyst notes, “[M]iddle power states are ideally suited to partner with NGOs in the pursuit of selected issues on the international agenda” [21].

These examples of global health diplomacy by middle owers are uncharacteristic of how traditional middle powers supposedly operate. Middle powers typically have a narrow band of foreign policy goals that they wish to achieve, but they mostly work within the multilateral system; they do not usually challenge the status quo or the rules of the game. Instead, they stabilize and legitimize the system and act as good international citizens that affirm the way things work. This is particularly true for traditional middle powers in the global north (e.g., Norway, Australia, and Canada).

In contrast, influential middle powers from the global south do in fact challenge the status quo—but they stop short of calling for revolution [22]. Countries such as Brazil, Mexico, South Africa, and Turkey may be interested in reforming rather than discarding the rules of the game. Such states have the potential to be the “norm entrepreneurs” that will push the envelope and call for reform, for altruistic as well as for selfish reasons: for instance, South Africa and Brazil agree that the UN is a dinosaur of the Cold War and that change is essential both to allow greater representation and to reflect contemporary international politics more accurately; at the same time, these states are interested in obtaining their own permanent seats on a reformed UN Security Council.

### South Africa’s (very) foreign policy

The fundamental tension between traditional and emerging middle powers identified above—namely, their role in affirming the status quo on the one hand while pushing to reform the global system on the other has been characteristic of South Africa’s foreign policy expression since the country democratized in 1994.

The Mandela government (1994–1999) emphasized the imperative for the former pariah state to integrate with the international community, and South African foreign policy during those years focused mainly on good international citizenship. At home, Mandela prioritized nation building and reconciliation, and abroad his government projected South Africa as a good, clean middle power with massive human rights capital [23]. South Africa did not challenge the global order by demanding any rewrite of existing international rules. Instead, Mandela’s Department of Foreign Affairs (DFA) was keen to be seen as a peacemaker on the African continent, a bridge builder between the global south and the global north, and a beacon of hope in a sea of injustice. South Africa was rewarded for its peaceful transition by being elected to chair multilateral bodies such as the Southern African Development Community (SADC) and the Non-Aligned Movement (NAM). Keenly sensitive to South Africa’s previous reputation as a regional bully during the apartheid regime, Mandela made a point to underplay the country’s status as the obvious regional and continental hegemon. Even when in 1998 a coup threatened the stability of the Kingdom of Lesotho, a sovereign state landlocked within South Africa, the Mandela government took pains to ensure SADC approval and stewardship of the eventual military intervention.

Whereas Mandela rarely ventured beyond rhetorical criticism of the injustices of the global order, his successor, Thabo Mbeki (1999–2008) had a distinctly different view. A former member of the Politburo of the South African Communist Party, Mbeki was a pragmatist who steered domestic economic, industrial, and trade policies towards liberalization. A keen intellect, Mbeki crafted his domestic and foreign policies based on ideas rather than simple opportunism. Foreign policy during the Mbeki era was an accommodation of a mosaic of ideological impulses. On the one hand Mbeki was a realist, aware that the country would be excluded from the benefits of globalization if it followed any policy trajectory that would scare off foreign investors. On the other hand he was an African Nationalist, using the lens of south-south or global southern solidarity to describe (and criticize) the way the world works [24]. Mbeki was fond of using the rhetoric of Marxist or structuralist thinking to apportion blame to the global north for their continuing imperial ambitions in Africa and in the global south more broadly. His Africanist tilt meant that he often rejected any intervention or criticism from the West in particular, insisting that Africans should seek African solutions to African problems.

As a result, South African foreign policy under Mbeki often seemed paradoxical [25]. The government insisted that it was guided by an African agenda with a strong human rights and a multilateral focus, while at the same time it increasingly acted to support counter-hegemonic regimes. For instance, during South Africa’s membership of the UN Human Rights Commission and its first two-year tenure as a non-permanent member of the UN Security Council (2007–2008), the country consistently blocked Western-led resolutions to condemn rape as a political or military weapon; it was opposed to the indictment of Sudanese president Omar al-Bashir as a war criminal and the push to bring him before the International Criminal Court in The Hague; and it repeatedly did not support resolutions condemning the regimes of Iran, Myanmar, and Zimbabwe for serious breaches of international law. South Africa seemed to talk the talk of human rights as its niche and its main driving force in foreign affairs, but it walked the walk of quixotic and blanket south-south solidarity to such an extent that it squandered much of its credibility as a champion of universal human rights [26].

This situation has not improved under Mbeki’s successor, President Jacob Zuma (2008–), who treats foreign relations in an insipid, business-as-usual way. Zuma’s foreign policy is what his diplomats make of it, and rhetorically it is a continuation of Mbeki’s priorities, which are, in order of importance, “the African agenda,” which has no clear definition; south-south cooperation; the centrality of multilateralism; north–south dialogue; and issues of global governance [27]. Foreign policy observers have noted that South Africa’s relations with the rest of the world are in need of a fresh motive; hollow notions regarding human rights and good international citizenship as stated in formal policy documents have become stale. In the words of one analyst, “There are countries that have established good reputations in their promotion of human rights in their foreign policy. But the flip side to this is that countries can lose their reputations. South Africa has certainly lost its reputation and practically all the gains of the Mandela era” [28].

The South African government itself identified the need for a renewed and coherent foreign policy focus. The DFA held a heads of missions meeting in February 2005 to consider strategic foreign policy priorities, and there was a consensus that the country should seek “innovative ways” to position itself as “an active agent of progressive change” [29]. It is clear that the DFA itself wanted to pursue an emerging middle power agenda that did not shirk from challenging existing orthodoxies and modes of global governance. In her analysis, Yolanda Kemp Spies, a former long-serving South African diplomat, updates this position by proposing for South Africa a more selective, *ad hoc* approach than it has thus far adopted in its multilateral projects. A more focused multilateral role will require rationalisation of foreign policy priorities. In establishing these, the government could draw on the expertise and “new policy ideas widely available in global civil society” by ensuring closer cooperation with transnational issue networks [30].

### Health in the new multilateralism

In the search for a niche and for improved agency for South African foreign policy, interesting themes emerge or are confirmed: “innovative” diplomacy, “transnational issue networks,” and “multilateralism” are clear; so too is an emphasis on the pursuit of issue diplomacy as a strategy available to emerging middle powers. There are certainly signs that South Africa’s foreign policy and the global political environment would accommodate such a shift, particularly in light of the emergence during the last decade of new south-south as well as north–south multilateralism: the India-Brazil-South Africa (IBSA) grouping was established in 2003; South Africa recently became the ‘S’ in the BRICS alliance that also includes Brazil, Russia, India, and China; and in 2008 the G-8 was replaced with the G-20 (a north–south grouping of the strongest international economies and “norm entrepreneurial” states). The question arises whether any or all three of these groupings might act as vehicles for exactly the kind of niche diplomacy role that South Africa seeks. Could health be the transnational issue that enables emerging middle powers in particular to pursue novel global networks and more achievable and sustainable foreign policy outcomes?

Existing opinions on this question are either silent on health specifically as a niche area for emerging middle power diplomacy, or they express tentative, qualified enthusiasm. The G-20 is viewed as an important grouping, but for the moment it is too new and unwieldy, and it may represent too many divergent members to facilitate the crafting of a coherent position on global health. At the moment the G-20 is most concerned with moderating the threats of the ongoing global financial crisis. However, the G-8 could serve as an example for the establishment in the G-20 of productive working groups on health issues specifically; this could be valuable in the pursuit of a shared consensus, first on health and development governance issues that do not elicit radically divergent points of view, and then gradually by engaging with issues of more serious disagreement.

In principle, most G-20 countries agree on many issues of global health governance, including the need to combat HIV, tuberculosis, and malaria; the prioritization of maternal and child health; and the imperative of improved aid efficiency and effectiveness. Potential G-20 working groups on these and other issues might build trust and also provide a forum for negotiation around more contentious issues [31]. Jorge Heine, a former Chilean ambassador to South Africa, warns that South Africa does not seem to realize the potential of a more “niche” focus within the G-20, and that the country does not “seem to have fully realised what the G-20 entails and ha[s] taken it rather nonchalantly” [32].

South Africa seems to view the IBSA grouping more seriously, and it has used it quite effectively as an emerging middle power coalition to challenge global rules around health. India is a prominent manufacturer of ARVs and other pharmaceuticals, and Brazil and South Africa have actively and successfully advocated for amendments to the TRIPS agreement, specifically to make ARVs available in their domestic environments. In doing so, IBSA has been successful at “soft balancing” that is, flexible coalition-building in global institutions which “does not directly challenge U.S. military preponderance, but rather uses non-military tools to delay, frustrate, and undermine the superpower’s unilateral policies” [33]. This strategy can also work for IBSA in other areas of global governance, as well as on other issues [34]. The trilateral alliance defines itself as a means towards broader cooperation among developing countries. They share a diagnosis of the failing health of established institutions of global governance, something recognised by the institutions themselves, and see in the application of regional representivity a means of re-legitimising these institutions, as well as of positioning themselves therein in a leadership role.

South Africa also appears to be more enthusiastic about its new BRICS membership than about the G-20. Yet, South Africa’s membership in this alliance seems to be more reflective of ideological solidarity than of practicality. The truth is that BRICS itself does not pay that much attention to health, beyond citing it as one among a range of issues that reflect the imperfections and injustices of the world. The most basic comparative metrics confirm that South Africa is an outlier in BRICS: its population, economy, and relatively low global status even as an emerging middle power render it the odd one out, and its membership has more to do with existing BRIC members’ consideration that they need an African member, as well as another demonstration of symbolic southern solidarity, than with South Africa’s diplomatic capabilities or status. For South Africa, one prominent attraction of membership is the opportunity to develop closer relations with China.

This does not mean that BRICS necessarily excludes the promise of health diplomacy. These three examples of multilateralism—IBSA, the G-20, and BRICS—all have the potential to improve global health. There are strong signals that IBSA in particular could make health diplomacy a mainstay of its international and transnational interactions. These organizations should seriously consider the various transformative roles that they can play: providing financial assistance for health projects and supplying medical goods and services to very poor countries in their immediate geographical neighborhoods; providing focused technical assistance in specific health niche areas; continuing to improve access to medicines and intellectual property, as there is already a history of significant success in this area; modeling effective health-sector framework-building to less developed countries; making an explicit decision to play a more proactive role in global health governance; and bolstering the link between health and foreign policy [35].

### AIDS diplomacy as a project of emancipation

In 1978, 134 representatives of WHO member states gathered in the Soviet Union and crafted the Alma Ata Declaration, calling for “Health for All by the Year 2000.” The declaration demanded “the attainment by all citizens of the world by the year 2000 of a level of health that will permit them to lead a socially and economically productive life” [36]. Two countries then embedded health as a fundamental human right in their constitutions: Brazil in 1988, as noted earlier, and South Africa in 1996. In 2000 the UN identified its Millennium Development Goals (MDGs), three of which are explicitly devoted to health. In 2007 the Oslo Ministerial Declaration underlined the need to integrate health in countries’ foreign policies.

This short history has an interesting trajectory; it reflects a growing Kantian impetus to apply power for good, for the pursuit of dignity for all, and to change the world for the better. At least that is the aspiration—an ambition that many states have imperfectly pursued. Health diplomacy has at long last been formalized on the global agenda and an opportunity exists for states and non-state actors to use a health lens to facilitate a transformative moment. According to critical theorists [37,38], international relations should be not only a field of study that describes the world as it is, but is also applied to enhance opportunities for emancipation. This includes freedom from want, allowing society to maximize its potential, and transforming the world and global governance to achieve dignity for all.

This is idealistic, but the principle is laudable. The application of a health lens in foreign policymaking might be one way in which emerging middle powers can find traction in the international system. But health is a broad field, with various normative underpinnings and contradictory agendas, domestically as well as globally. The established notion of niche diplomacy begets the identification of transnational issue networks that can be used to advocate for improved health outcomes. To that end, South Africa can and should explore AIDS diplomacy within the broader perspective of health diplomacy to reclaim the sense of purpose in its foreign policy, to salvage the human rights focus as well as the moral stature that it seems to have lost.

#### Why AIDS diplomacy?

Nearly six million South Africans are HIV-positive; this constitutes one in every five members of the country’s adult population. Clearly, the South African AIDS epidemic is an exceptional event that will define much of the country’s image and demand a great deal of its policy and fiscal attention for many years to come.

Many in the country feel a sense of outrage that the “Rainbow Nation” survived the horror of apartheid only to be ravaged by such an insidious virus in the infancy of its democracy. Adding insult to injury, the country’s history of managing the epidemic is an anthology of some of the most pathological elements of African governance: the apartheid government (1948–1994) ignored the epidemic, welcoming it as a way to cull those deemed “undesirable” in South African society: gays, blacks, prostitutes, and drug users. The Mandela government (1994–1999) likewise looked elsewhere, concentrating on consolidating democratic institutions and nation building. Possibly worse yet, the Mbeki administration (1999–2008) was accused of AIDS denialism, blaming the viral syndrome on the structural and imperial ambitions of the West and refusing to make ARVs available to millions of citizens [39]. In the process, between 2000 and 2005 more than 330,000 South Africans suffered and died preventable deaths, and more than 50,000 infants were born HIV-positive because the government prevented their mothers from accessing medication that would have restricted postnatal transmission of the virus [40]. Max Essex of Harvard University refers to South Africa’s AIDS policy during the Mbeki years as “a case of bad, or even evil, public health” [41].

Yet, since Mbeki’s departure and South Africa’s AIDS policy rehabilitation in 2008, the country has failed to take up global leadership on HIV/AIDS. There is excellent precedent for South Africa to capitalize on its ability to overcome dread, however: after apartheid, the country proactively cultivated an image of itself as a nonracial and tolerant society, and it worked hard to project this image via its human rights-focused foreign policy. Unfortunately, the current South African government has thus far chosen to ignore the lessons of AIDS that it could share with the world; HIV and AIDS do not receive special mention in the country’s foreign policy as an issue that can be used to enable conversations about broader challenges of global health governance. This is a missed opportunity.

The irony is that South Africa had been successful at AIDS diplomacy not that long ago. During the Mandela and first Mbeki administrations, several important pieces of legislation were introduced and case law came to fruition that further served to entrench a human rights approach to HIV and AIDS. The *Medicines Act* of 1997 was the first and had the highest media profile, domestically and internationally. In brief, the act contained provisions that would make it possible for the South African government to parallel import ARVs from abroad and to issue compulsory licenses to local pharmaceutical manufacturers to produce generic AIDS drugs at a fraction of what it would cost to purchase such drugs from their foreign patent holders. Such measures are legal under the WTO TRIPS rules (of which South Africa is a signatory), but the *Medicines Act* became controversial when the Pharmaceutical Research and Manufacturers of America (PhRMA), representing 39 pharmaceutical companies, decided in 1998 to take the Mandela government to court on this issue.

During the second half of the Mandela administration, the Clinton administration placed immense pressure on South Africa to honor the patent rights of the American companies that developed the drugs, going so far as to put South Africa on the U.S. “Super 301” trade watch list, which according to the U.S. Trade Act of 1974 authorizes the President to take all appropriate action, including retaliation, to obtain the removal of any act, policy, or practice of a foreign government that violates an international trade agreement or is unjustified, unreasonable, or discriminatory, and that burdens or restricts U.S. commerce. Many observers accused the U.S. government of using bullying tactics to put profits above the rights of people living with the virus, and eventually the issue became a political and public relations disaster for the U.S. government.

The South African government (both under Mandela and in the early years of the Mbeki administration) did manage, however, to put on the international agenda the issue of HIV-positive people’s rights to essential drugs. The issue also garnered wide support from sectors of civil society; the TAC and the trade union COSATU in particular established links in South Africa and abroad in the global south as well as the global north—to drive popular resistance and demonstrations against the PhRMA court action. In April 2001 PhRMA decided to withdraw its case against the South African government; South African and international AIDS communities hailed this move as a victory for human rights in the context of HIV and AIDS. South Africa had triumphed in a David versus Goliath battle against big business. However, the elation was short-lived: at the news conference following PhRMA’s decision to withdraw its legal challenge, the South African minister of health announced that the drugs remained too expensive to parallel import or to manufacture locally.

Although South Africa did not fully capitalize on its triumph, the PhRMA case did result in several interesting foreign policy actions. First, the South African government focused on a single issue that it pursued robustly in front of domestic as well as international audiences. Additionally, a transnational issue network consisting of state and non-state actors cooperated to make victory possible. Finally, in the process, South Africa achieved success, not only when the pharmaceutical companies abandoned their case and the country was removed from the American trade watch list, but also at the WTO where, in November 2001, it advocated successfully with Brazil and other states to amend the TRIPS regime on pharmaceuticals. This is an excellent example of emerging middle powers cooperating with broader issue networks in an instance of health diplomacy not to challenge an entire global governance model, but to reform health multilateralism.

Unfortunately, South Africans have thus far lacked the will to rekindle their moral leadership on global AIDS governance. If they chose to do so, this might go some way not only to redeem Mbeki’s genocidal AIDS policies at home, but also to rehabilitate the country’s foreign policy stature. Such a move could serve an important ideological purpose, and instead of simply accepting AIDS policy “best practice” from the global north, it could boost South Africa’s status as a middle power that focuses on reforming rather than affirming imperfect health governance globally. South African AIDS diplomacy might become a metaphor for transformation; it could come to represent a broader project of emancipation, not only regarding health inequalities, but also on questions of justice and equity globally. This would move the country’s foreign policy rhetoric beyond the level of anachronistic Marxist or dependency theories that contradict South Africa’s free-market macroeconomic and trade policies.

#### A suggested agenda for AIDS diplomacy

Six broad areas or sets of issues could frame an emerging AIDS diplomacy in South Africa. This list should be prefaced with the observation that the development of such niche diplomacy in itself could strengthen South African democracy. The post-Mbeki foreign policy discourse in South Africa is slight, and a serious and focused conversation in the epistemic and policy communities in itself would be a worthwhile consequence of the development of new foreign policy values and options.

It is imperative that such an exercise, particularly in the case of this epidemic, should include members of South Africa’s AIDS civil society. Just as the government set an important AIDS diplomatic precedent with the PhRMA case in the late 1990s, so the experience of TAC and its global issue alliance partners would provide invaluable insights into the kind of nongovernmental and transnational diplomacy that it practiced in the darkest days of Mbeki’s AIDS denialism. In addition to the possible development of an inclusive definition of South African AIDS diplomacy, this would also help to heal the rifts that might still exist due to the antagonistic positions of the former administration on the one hand and AIDS civil society on the other.

The first area of attention for AIDS diplomacy is global trade, as “[the] trade and health linkage highlights the new prominence of health within foreign policy” [42]. As discussed above, both Brazil and South Africa have strong records of achieving transformation in existing multilateral trade rules regarding pharmaceuticals in particular. In addition, transnational issue networks from both countries have achieved remarkable progress in negotiating lower prices for ARVs. South Africa should advance its trade agenda with a specific focus on the facilitation of technology transfer, both north–south and south-south.

The second agenda item for the application of an AIDS diplomacy lens pertains to South Africa’s status as an emerging middle power. Because this is a status that the country wishes to entrench and build upon, it should explore closer multilateral ties with Brazil (via IBSA) as well as with other emerging middle powers, and it should seek to align common goals for united health diplomacy. Such an initiative would reflect positively on middle powers’ view that sovereignty is responsibility. Issues that could be explored at the level of AIDS diplomacy multilateralism include the transformation of the existing architecture of global health governance. Sustained and concerted public diplomacy and advocacy within the World Health Assembly, the body that governs the WHO, could be productive. This would demonstrate middle powers’ ability to set the official agenda, instead of simply affirming the list of priorities that emanates from Geneva and New York.

Such an amended agenda should emphasize agency in strengthening health systems in middle and low-income states and highlight the need for sustainable funding of health initiatives in poor countries. In engaging at the multilateral level in this way, a country like South Africa could give voice to those who are normally excluded from such fora, including the elderly, women, children, and people living with HIV. In the context of acting to channel the voice of the disenfranchized, it is important that activist states cooperate and consult in the first instance with transnational activism networks in the global south, rather than automatically following the ostensibly benign agenda of civil society in the global north [43].

Third, South Africa could follow the example of South American states and set up a regional institution focusing on health issues within SADC. “The Pan-American Health Organization (PAHO) […] serves as a platform for coalition-building and regional initiatives for Brazilian pharmaceutical diplomacy” [44]. A pan-African organization that is devoted to health issues more exclusively might do better than subsuming health as one of many policy areas at the SADC Secretariat. Given China’s stated enthusiasm to expand its interests and influence on the African continent, South Africa might request funding for such an organization from the Chinese government, or it may form a consortium of southern states via BRICS or IBSA. Brazil could also play a role in mentoring Southern African in its management of and strategy for such a regional health body. One issue that could be championed by South Africa within such a forum is the expansion of the rights of minority groups against whom there is increasing discrimination in Sub-Saharan Africa. Gays and lesbians in particular are persecuted in the SADC, and South Africa would do well to share the lessons of its human rights approach with sexual minorities.

Fourth, in addition to multilateral and regional AIDS diplomacy, South Africa should also strengthen “catalytic” diplomacy, which refers to multiple interactions between diplomatic actors that “create a sort of symbiosis between state and non-state activities” [45]. Such an initiative could reinforce and expand alliances between members of domestic and foreign or transnational civil society. Transnational issue networks play an increasingly critical role as global norm entrepreneurs, and this function should be cultivated. As the executive director of UNAIDS and his colleagues put it, “political momentum can be built by linking HIV-issue specific networks with movements that seek equity, justice and fairness in relation to other concerns—be they related to funding, trade, access to technology or climate change” [46].

The fifth area in which AIDS diplomacy could be beneficial is in training and retaining health personnel, and addressing the causes of the unequal global distribution of doctors and (especially) nurses. This is a critical area for the management of AIDS and other infectious diseases, and at the moment the global south loses thousands of its medical personnel to the global north every year. Africa has 2.3 health workers per 1000 population, whereas in the Americas there are 24.8 healthcare workers per 1000 [47]. Any initiative to address this issue should reflect on both the push and the pull factors—facile condemnation of rich countries’ recruitment practices should not blind governments in the global south to those determinants of health personnel migration that are within their control. South Africa might start such an initiative by pushing for progress on developing more advanced international codes of conduct on the recruitment of health personnel. Specifically in the context of the AIDS pandemic, South Africa could facilitate initiatives to strengthen the health workforce by exploring innovations around task-shifting to deliver HIV services [48].

Finally, South Africa can take the lead in training health diplomats. During June 18–22, 2007, the Graduate Institute of International Relations in Geneva ran its first Summer Program on Global Health Diplomacy, and it would be fairly simple to build a health/AIDS diplomacy element into the existing training that diplomats in Pretoria receive. Such training is essential, as “[h]ealth diplomacy requires new interdisciplinary approaches for health sciences education to prepare professionals who will manage persistent and emerging global heath challenges” [49]. The training can also be made available to the diplomatic trainees of other states.

At the moment the University of Pretoria is the only Southern African tertiary education facility that has a formal postgraduate program on diplomacy as a distinct field of study. This can be expanded to other institutions, and a curriculum on AIDS and health diplomacy could include key areas of interest such as clinical diplomacy (particularly in non-Western settings), health security, human rights, social justice and equity, health manpower, global science, global economics, and changing aid agendas.

#### Some caveats

With any opportunity there are risks, and if South Africa decides to develop a nascent AIDS diplomacy it should be aware of a few fairly obvious potential hazards. First, avoid an agenda that is conceptualized in excessively broad or ambitious terms; keep AIDS diplomacy or health diplomacy more generally practicable and take care not to make the mistake of trying to do too much too soon. Additionally, resist the temptation to securitize health or AIDS as policy issues. Political science and international relations courses are hardwired with a state-centric and Realist perspective, and this might trickle into understandings of all human security issues. As discussed above, health diplomacy is not the same as health security.

South Africa’s AIDS policy rehabilitation since 2008 has led to many constructive changes. The country has been remarkably successful with its test-and-treat program, and the denialism of the Mbeki era truly seems to be a thing of the past, at least at the level of government. Yet, AIDS diplomacy should not lead to a wholesale embrace of the current medical triumphalism, or of “best practice” models that have been developed in different contexts.

Lastly, niche or issue diplomacy is exactly that; it should not overtake the overall foreign policy directives of any state. South African foreign policy cannot only be about health and AIDS; the latter should be complementary to a broader strategy and evolving consensus about the kind of state and society that South Africa wants to be, and how it wishes to project itself beyond its borders.

As such, the national department of health should not be the institutional custodian of South Africa’s foreign policy—the point is that health and HIV/AIDS cut across all levels and departments of government. Rather, AIDS diplomacy should reside at a cross-departmental level, most probably in the president’s office.

### Summary

Instrumentally, South Africa can cultivate a foreign policy motive theme around Global Health in general and AIDS specifically to regain some of the soft power and credibility that the country has lost due to the Mbeki administration’s AIDS denialism. But why would other states believe this, or think that South African AIDS diplomacy is worth emulating? The short answer is that institutionally, historically as well as in terms of precedent, the makings of AIDS diplomacy as a feature of South African foreign policy are already in place. There are four broad theses in support of such a statement:

Firstly, in September 2006 the country was a founding member of the Global Health and Foreign Policy Initiative, which explicitly frames health as the most important long-term foreign policy issue of our time. Secondly, South Africa is one of very few states which enshrines the Right to Health in its Constitution. Thirdly, the country is exemplar of significant and recent precedent in the use of transnational issue networks for global change: (1) institutional memory remains strong with regards to the defeat of apartheid by means of catalytic and public diplomacy, and through the accrual of soft power; (2) after global pharmaceutical companies cynically challenged the new South African *Medicine’s Act* in 1997, in April 2001 a global campaign driven by a right-to-health ethos and a supportive, progressive Constitution led to the defeat of these big PhRMA; (3) in November 2001 Brazil and South Africa worked with other emerging middle powers and transnational civil society campaigners to successfully challenge the existing TRIPS Agreement specifications regarding the availability of pharmaceuticals in poor countries that experience epidemic distress; (4) the country’s foreign policy on paper as well as in practice is strongly in favour of progressive and radical change to the status quo. Lastly, South Africa has already demonstrated significant HIV/AIDS policy and practice rehabilitation since the departure of President Thabo Mbeki: it now manages the largest national test-and-treat AIDS programme in the world, and it has embraced AIDS policy good practice emanating from the World Health Organization. A country which radically challenged and ultimately defeated its nefarious racist policies in the past has now also rehabilitated its AIDS management—why should the outside world praise the country for the former, but not for the latter?

The Oslo Ministerial Declaration on the link between health and foreign policy has opened a window of opportunity for a concerted effort to mainstream health as an issue to be used not only to affirm the status quo of global health governance, but also to reform the health architecture and to consider the kind of global order that might be possible. Similarly, AIDS diplomacy can be a project of transformation and emancipation for South Africa, which is in need of a refocused and rejuvenated foreign policy. The country has overcome dread before, transforming the legacy of apartheid into an example for the rest of the world to admire. By exploring innovative diplomacy, South Africa can use AIDS as an issue to consolidate its young democracy, and it can work with transnational issue networks to strengthen middle power multilateralism more broadly. If South Africa can get this right, it has the real potential to turn dread into capital, rather than to default to positions of impotence and blaming outsiders.

## Competing interests

An amended version of this debate piece was published as an online report commissioned by the Africa Program and Global Health Policy Center at the Center for Strategic and International Studies in Washington, DC. The original report can be downloaded here: http://csis.org/publication/turning-dread-capital. The author has requested and received the necessary written permission to republish this amended version of the report. The author further declares that his current employer, Stellenbosch University, finances the article-processing charge.

## Authors’ information

Department of Political Science, Stellenbosch University, Private Bag X1, Matieland 7602, South Africa.
